# Regulation of Epigenetic Modifiers, Including KDM6B, by Interferon-γ and Interleukin-4 in Human Macrophages

**DOI:** 10.3389/fimmu.2017.00092

**Published:** 2017-02-08

**Authors:** Gökçe Yıldırım-Buharalıoğlu, Mark Bond, Graciela B. Sala-Newby, Charles C. T. Hindmarch, Andrew C. Newby

**Affiliations:** ^1^Chair of Vascular Cell Biology, School of Clinical Sciences, University of Bristol, Bristol, UK; ^2^Department of Biomedical and Molecular Sciences, Queen’s University, Kingston, ON, Canada; ^3^Department of Physiology, Faculty of Medicine, University of Malaya, Lembah Pantai, Kuala Lumpur, Malaysia

**Keywords:** macrophage phenotype, epigenetics, interferon-γ, interleukin-4, proliferation

## Abstract

**Background:**

Interferon-γ (IFN-γ) or interleukin-4 (IL-4) drives widely different transcriptional programs in macrophages. However, how IFN-γ and IL-4 alter expression of histone-modifying enzymes involved in epigenetic regulation and how this affects the resulting phenotypic polarization is incompletely understood.

**Methods and results:**

We investigated steady-state messenger RNA levels of 84 histone-modifying enzymes and related regulators in colony-stimulating factor-1 differentiated primary human macrophages using quantitative polymerase chain reaction. IFN-γ or IL-4 treatment for 6–48 h changed 11 mRNAs significantly. IFN-γ increased CIITA, KDM6B, and NCOA1, and IL-4 also increased KDM6B by 6 h. However, either cytokine decreased AURKB, ESCO2, SETD6, SUV39H1, and WHSC1, whereas IFN-γ alone decreased KAT2A, PRMT7, and SMYD3 mRNAs only after 18 h, which coincided with decreased cell proliferation. Rendering macrophages quiescent by growth factor starvation or adenovirus-mediated overexpression of p27^kip1^ inhibited expression of AURKB, ESCO2, SUV39H1, and WHSC1, and mRNA levels were restored by overexpressing the S-phase transcription factor E2F1, implying their expression, at least partly, depended on proliferation. However, CIITA, KDM6B, NCOA1, KAT2A, PRMT7, SETD6, and SMYD3 were regulated independently of effects on proliferation. Silencing KDM6B, the only transcriptional activator upregulated by both IFN-γ and IL-4, pharmacologically or with short hairpin RNA, blunted a subset of responses to each cytokine.

**Conclusion:**

These findings demonstrate that IFN-γ or IL-4 can regulate the expression of histone acetyl transferases and histone methyl transferases independently of effects on proliferation and that upregulation of the histone demethylase, KDM6B, assists phenotypic polarization by both cytokines.

## Introduction

Macrophages are important, multifunctional cells in the innate immune system. Their ability adopt a spectrum of phenotypes that perform greatly different functions in response to diverse activators has become increasingly recognized (1). IFN-γ is an important pro-inflammatory cytokine in responses to certain pathogens, promoting toll-like receptor expression and inducing greater production of nitric oxide, pro-inflammatory cytokines, such as TNFα (2, 3) and extracellular proteases, including some metalloproteinases (4), which together promote invasion of macrophages to sites of inflammation and enhance microbial killing. IFN-γ is also an established link between the innate and acquired immune systems, especially in the context of autoimmunity, where it not only promotes activation of Thelper1 lymphocytes but also increases major histocompatibility complex (MHCII) expression leading to enhanced antigen presentation by macrophages (3). Macrophages activated by IFN-γ are believed to provoke tissue injury, for example joint destruction during rheumatoid arthritis and atherosclerotic plaque rupture leading to myocardial infarctions (5). On the other hand, IL-4 or IL-13 provoke a macrophage phenotype that has greater scavenger receptor activity and increased release of anti-inflammatory and fibrogenic factors (6, 7), suggesting a primary role in clearance of cell debris and promotion of tissue repair. Although apparently more benign, these macrophages may help tumor cells evade immune surveillance and can provoke allergy or lung hypersensitivity (6, 7). Greater understanding of the mechanisms that underlie generation of these diverse macrophage phenotypes is, therefore, warranted in order to design strategies to avoid these unwanted complications.

Responses to IFN-γ are mediated through signal transducer and activator of transcription-1 and several so-called interferon response factors (IRFs) (8–10), especially IRF-7 and IRF-9 (1), whereas IL-4 and IL-13 activate STAT-6 (11) and IRF-4 (1). Consequently, IFN-γ and IL-4 provoke widely different transcriptional responses, effectively defining their divergent phenotypes (12). Moreover, the ability of IFN-γ to downregulate many IL-4-induced genes [for example cluster E vs F of reference (12) and module 15 of reference (1)] amplifies these phenotypic differences. Participation of epigenetic mechanisms in macrophage polarization has also been demonstrated (13). In particular, the ability of IFN-γ or IL-4 to alter the local histone code, which determines whether the relevant transcription factors have access to promoter sequences, has been implicated in their ability to drive cells toward different phenotypes (14–17). However, knowledge regarding the role of specific histone-modifying enzymes is presently fragmentary and sometimes conflicting (13). We, therefore, took an unbiased approach by using an RT-qPCR array of 84 epigenetic regulators to investigate the impact of IFN-γ and IL-4, singly and in combination (to look for antagonistic effects) on human primary macrophages. We identified 11 genes up- or downregulated by the cytokines but some of these were affected, at least in part, secondary to inhibition of cell proliferation. Lysine demethylase 6B (KDM6B) was the only putative activator of transcription that was upregulated by both IFN-γ and IL-4, thereby implying a functional role in promoting gene expression by both cytokines. To investigate this hypothesis directly, the functional consequences of inhibiting and silencing KDM6B were investigated further.

## Materials and Methods

### Cell Preparation

Monocytes were isolated from the EDTA anticoagulated blood of healthy volunteers. Written informed consent was given under National Research Ethics Service approval from Frenchay Research Ethics Committee reference 09/H0107/22 and South West 4 Research Ethics Committee reference 10/HO102/72, respectively. Mononuclear cells were isolated using Ficoll-Paque Plus (GE Healthcare Life Sciences), red blood cells were lysed with 150 mM ammonium chloride/0.1% BSA, and monocytes were allowed to adhere to plastic in RPMI 1640/1% human serum AB (SigmaAldrich) for 1 h. Non-adherent cells were removed with warm Dulbecco’s phosphate buffered saline (D-PBS; Gibco), and the medium was replaced with RPMI 1640/10% fetal bovine serum (FBS; SigmaAldrich) for 1 h. Based on staining with Rabbit anti-(human CD14) antibody, Ab78313, adhered cells were at least 85% monocytes. Monocytes were differentiated into macrophages in RPMI 1640 medium containing 10% FBS and 20 ng/mL of colony-stimulating factor-1 (human recombinant CSF-1, R & D systems), which was replenished on day 3. Approximately 80% of the resulting cells were macrophages based on positive staining with mouse monoclonal anti-CD68 (M0876, Dako). Differentiated macrophages were treated for 6, 18, 32 and 48 h in the same medium with either 100 ng/mL of recombinant human interferon-γ (IFN-γ) (R & D systems) or 10 ng/mL of recombinant human interleukin-4 (IL-4) (R & D systems). AZD1152 was purchased from Selleck.

### RNA Isolation, Reverse Transcription, and Transcript Quantification

Total RNA was isolated from the macrophages prepared from three different healthy donors using the PureLink™ RNA Mini Kit (Ambion). Total RNA was quantified using an ND1000 NaNo Drop spectrophotometer, and 100 ng were reversed transcribed using the QuantiTect Reverse Transcription Kit (Qiagen) with additional genomic DNA elimination step indicated in the manufacturer’s instructions. For quantitative polymerase chain reaction (qPCR), the cDNA samples were diluted 1:3 in 10 mM Tris–HCl, pH 8.0 and amplified using the LightCycler 480 SYBR Green I Master mix (Roche) in an Eco Real-Time PCR System (Illumina), using primer sets shown in Table 1. Data were normalized to total RNA in each reaction. For qPCR array, RNA from three different donors was analyzed using the Human Epigenetic Chromatin Modification Enzymes RT^2^ Profiler PCR Array (Qiagen) according to the manufacturer’s protocol. Briefly, total RNA (400 ng, genomic DNA eliminated) was reverse transcribed using RT^2^ First Strand Kit (Qiagen) and diluted in RNase-free water. The amplification reaction was conducted in 384 well format in a Roche LightCycler 480 (95°C 10 min for one cycle followed by 95°C 15 s and 60°C 1 min for 45 cycles). Threshold cycle (C_T_) values were exported and analyzed using web based SABiosciences PCR Array Data Analysis Software.^1^ A panel of five housekeeping genes integral to the array were used to calculate for each probe ΔC_T_ = (C_T_ probe − C_T_ average of housekeeping genes). The values of ΔΔC_T_ = ΔC_T_ experimental sample − ΔC_T_ control were calculated for each probe and converted to fold changes = (2^(−^Δ^ΔCT)^). For transcriptomic analysis, purified RNA samples from four different donors were submitted to the Illumina Gene Expression ServiceXS (Leiden, Netherlands) and were processed for analysis on the Illumina HumanHT-12 v4 microarray as described in detail.^2^ The results were deposited under number GSE83957. Fold changes and statistics (multiple testing corrections) of generated raw data were performed using GeneSpring (Agilent Technologies). Venn Diagrams were generated by using web-based software.^3^ Gene ontology enrichment (GOE) and KEGG pathway analysis were performed using the DAVID public database.^4^

**Table 1:** **Primers used for RT-qPCR**.

Gene	Sequences for PCR from 5′ to 3′	
AURKB	Forward	AGTGCCTTGGACCCCAGCTCTC
	Reverse	GTGACAGGCTCTTTCCGGAGGACT
CCL7	Forward	CCAACATGAAAGCCTCTGCAGCAC
	Reverse	TCTGTAGCTCTCCAGCCTCTGCTT
CD206	Forward	CGGTGACCTCACAAGTATCCACAC
	Reverse	TTCATCACCACACAATCCTCCTGT
CIITA	Forward	GGAGGCTTATGCCAATATCGCGGA
	Reverse	CCCAACTTCTGCTGGCATCTCCAT
CYCLIN E	Forward	CGCAGGGAGCGGGATGCGAA
	Reverse	CCGTCCTGTCGATTTTGGCCATTTC
ESCO2	Forward	TGCAGAACCCATCAAACAGGCATT
	Reverse	ATTGCCAAGCCCTAGGACATTCCG
KAT2A	Forward	CTCGGCTTGCAAGGCCAATGAAAC
	Reverse	CTCCAAGTGGGATACGTGGTCAGC
KDM6A	Forward	CCATGAACACAGCACAGCAGGCAT
	Reverse	CTTGGCAGGACTGGACAGGTCATC
KDM6B	Forward	GCAACCACCGCCTGCGTGCCTTAC
	Reverse	CGGGAATGCCTGGGTTCGGCTCCA
NCOA1	Forward	TGGGTTGCCTCTTCATTTACAGGG
	Reverse	TGGCTTCAGGGATGCTTTATTATCCT
SOCS3	Forward	CCCCCAGAAGAGCCTATTACATCT
	Reverse	GTACTGGTCCAGGAACTCCCGAAT
PALLD	Forward	GTATAAAGCCCGATACCTGCCCCG
	Reverse	CTGGAGTTGCTGGAGCTTCAGAGG
PCNA	Forward	CATGGGCGTGAACCTCACCAGTATG
	Reverse	ATACTAGCGCCAAGGTATCCGCGT
PRMT7	Forward	TTCCAGTTCTGCTTTAGGACCCGC
	Reverse	CCTCCGCTGCTACCACTTTTACCG
SETD6	Forward	CGAGGAAACGCGCTCTTAGACCA
	Reverse	CTCGCTCACCTTGGGACTCAGCTC
SMYD3	Forward	GATGGAGCCGCTGAAGGTGGAAAA
	Reverse	CCAAGGGATCCGAGCGGAAGAGTA
SUV39H1	Forward	ATAGACAACCTTGACGAGCGGCTG
	Reverse	ACGGGGTCCACTTGCATGTTGTAA
VAMP5	Forward	GGTGGTTGGTGTCCTGCTCATCAT
	Reverse	CTTCAGGACCAGCTGGGTCAGTTC
WHSC1	Forward	GATGCGACGCACCGCAGTGTTCTA
	Reverse	CCGAGGATTTCTGGTGCCTGCTT
36B4	Forward	GCCAGCGAAGCCACGCTGCTGAAC
	Reverse	CGAACACCTGCTGGATGACCAGCCC

### Western Blotting

Macrophages were lysed in SDS lysis buffer [2% SDS (w/v)/16% glycerol (v/v) in 50 mM Tris, pH 6.8]. Protein was measured (Micro BCA kit, Thermo Scientific Pierce). Equal amounts of reduced protein were fractionated by SDS-polyacrylamide gel electrophoresis, transferred to PVDF membranes (Merck Millipore), blocked in TBST/5% skimmed milk followed by incubation in primary antibody. Proteins were detected using appropriate HRP-conjugated secondary antibodies (SigmaAldrich) and enhanced chemiluminescence (Immobilon, Merck Millipore) and Hyperfilm™ ECL (GE Healthcare Life Sciences). The antibodies used were Phospho-Rb-Ser807/811, E2F1, KDM6A, and SMYD3 (Cell Signaling), Histone H3 and Histone H3-Phospho S10 (Abcam), GAPDH (Millipore), and p27^kip1^ (BD Biosciences).

### Macrophage Proliferation

To measure S-phase entry to the cell cycle, macrophages were labeled with 10 µM BrdU (SigmaAldrich) for 24 h. Cell proliferation was quantitated by immunohistochemistry as previously described (18). The percentage of BrdU positive nuclei was counted using NIH ImageJ software.

### Recombinant Adenoviruses and Infection of Macrophages

Recombinant adenovirus encoding for human p27^kip1^ was a kind gift from Professor Betsy Nabel (NIH, MD, USA). This virus and a control adenovirus expressing destabilized, enhanced green fluorescent protein (dsEGFP) were used as described previously (19). To generate the adenovirus expressing E2F1, the plasmid E2F1 wt-pGex2TK containing the coding sequence for human E2F1 from William Kaelin (20), was purchased from Addgene (Addgene plasmid # 21668). It was amplified using KOD DNA polymerase (Merck-Millipore, UK) to include *Eco*RI and BamH1 flanking sites and subcloned into the shuttle vector pDC515io from Microbix (ON, Canada). Recombination (Flp/FRT mediated) was performed in 293IQ cells to inhibit transgene protein expression (a gift from Dr. D. Matthews, University of Bristol) (21). For gene silencing, short hair pin (Sh) sequences were predicted using http://cancan.cshl.edu/RNAi_central/RNAi.cgi?type=shRNA. The sequences used to silence KDM6A, 5′CTGCCATTAAATGCTACTTAAATAGTGAAGCCACAGATGTATTTAAGTAGCATTTAATGGCAT3′, KDM6B, 5′CGCCCAGTCTGTGAAACCGAAGTAGTGAAGCCACAGATGTACTTCGGTTTCACAGACTGGGCA3′ and firefly luciferase, 5′CGCCTGAAGTCTCTGATTAATAGTGAAGCCACAGATGTATTAATCAGAGACTTCAGGCGGT3′, as a control, were embedded in the backbone of the primary microRNA-30, as described previously (22) and were synthesized by Eurofins. DNA sequences for modified microRNA-30 were synthesized by Eurofins, cloned into the Nhe1-BamH1 sites of the shuttle vector pDC515 and adenoviruses were generated as described above. Virus stocks were purified by CsCl banding and titrated by plaque assay. Monocytes differentiated for 4 days were infected with Ad-p27^kip1^ or Rad66 at 10^8^ plaque forming units (pfu)/ml for 24 h. Alternatively, cells were infected with Ad-E2F1 or Ad-dsEGFP at 5 × 10^7^ pfu/mL for 20 h followed by further 18 h treatment in the presence of IFN-γ or IL-4. For gene silencing, cells were infected with shKDM6A, shKDM6B, or shLuciferase (shLUC) viruses at 2 × 10^8^ pfu/ml for 72 h before stimulation with IFN-γ or IL-4 for 6 h.

### Statistical Analysis

Normality of data sets was analyzed by the method of Kolmogorov and Smironov. Differences between means of normally distributed variables with similar variances were analyzed using a paired Student’s *t*-test or, for multiple comparisons, ANOVA followed by a Dunnett or Student–Newman–Keuls post-test, as appropriate. For the array, data from the Benjamini–Hochberg method were used for multiple testing correction. All data are from independent experiments on cells from different donors and are presented as mean ± SE. *indicates *p* < 0.05, **indicates *p* < 0.01, ***indicates *p* < 0.001.

## Results

### Effects of IFN-γ and IL-4 on mRNA Levels of Epigenetic Regulatory Genes

**Figure 1:**
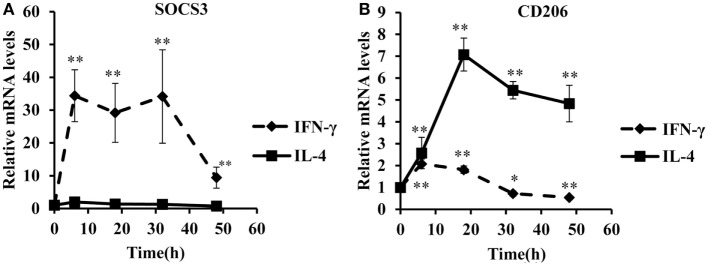
**Time course analysis of SOC3 and CD206 mRNA in response to interferon-γ or interleukin-4 (IL-4)**. Time course analyses of mRNA levels (RT-qPCR) of SOC3 and CD206 mRNA in response to by IFN-γ or IL-4 treatment of 4-day differentiated human monocyte-derived macrophages. **(A)** SOC3 and **(B)** CD206. Results are expressed as mRNA relative to time 0 untreated control. IFN-γ (solid line) and IL-4 (dashed line). Data are the mean ± SEM, *n* = 3 blood donors. *p* Values were calculated using ANOVA with Dunnett post-test. *indicates *p* < 0.05, **indicates *p* < 0.01 compared with control.

**Figure 2:**
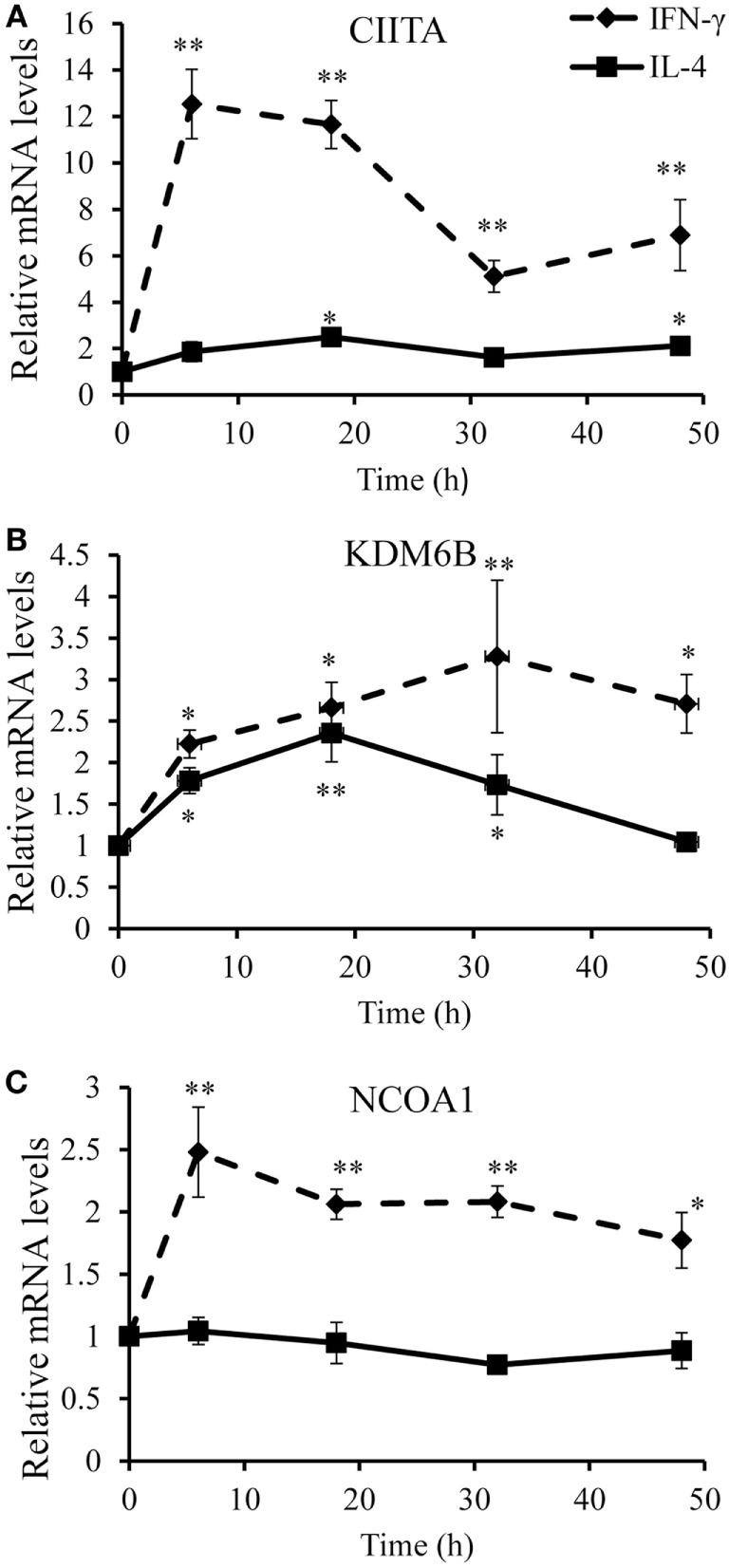
**Validation of upregulated genes**. Time course analyses of mRNA levels (RT-qPCR) of genes upregulated by IFN-γ or interleukin-4 (IL-4) treatment of 4-day differentiated human monocyte-derived macrophages. **(A)** CIITA, **(B)** KDM6B, and **(C)** NCOA1. Results are expressed as mRNA relative to time 0 untreated control. IFN-γ (solid line) and IL-4 (dashed line). Data are the mean ± SEM, *n* = 3 blood donors. *p* Values were calculated using ANOVA with Dunnett post-test. *indicates *p* < 0.05, **indicates *p* < 0.01 compared with control.

**Figure 3:**
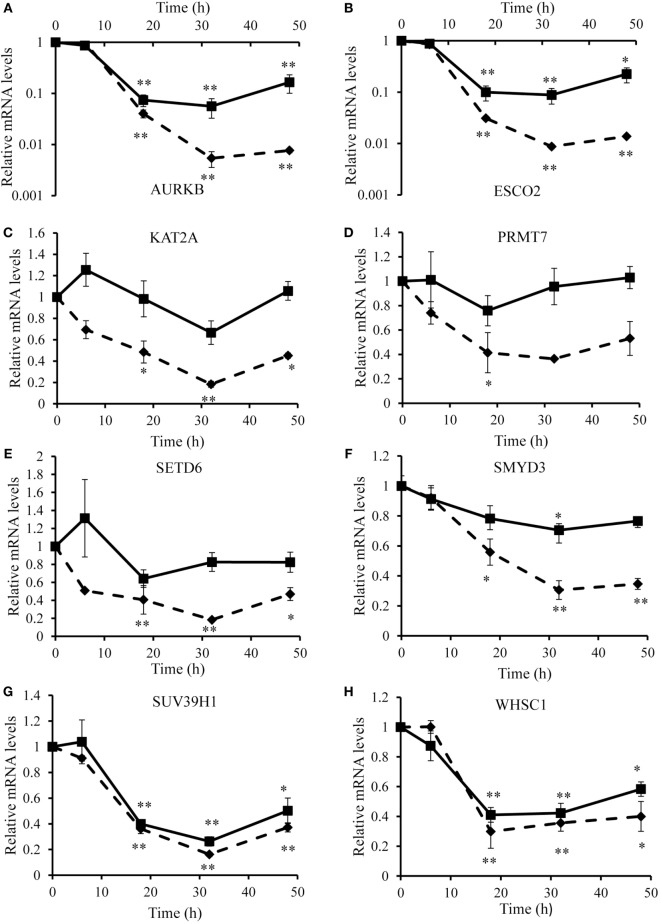
**Validation of downregulated genes**. Time course analyses of mRNA levels (RT-qPCR) of genes downregulated by IFN-γ and/or interleukin-4 (IL-4) treatment of 4-day differentiated human monocyte-derived macrophages. **(A)** AURKB, **(B)** ESCO2, **(C)** KAT2A, **(D)** PRMT7, **(E)** SETD6, **(F)** SMYD3, **(G)** SUV39H1, and **(H)** WHSC1. Results are expressed as mRNA relative to time 0 untreated control. IFN-γ (solid line) and IL-4 (dashed line). Data are the mean ± SEM, *n* = 3 blood donors*. p* Values were calculated using ANOVA with Dunnett post-test. *indicates *p* < 0.05, **indicates *p* < 0.01 compared with control.

**Figure 4:**
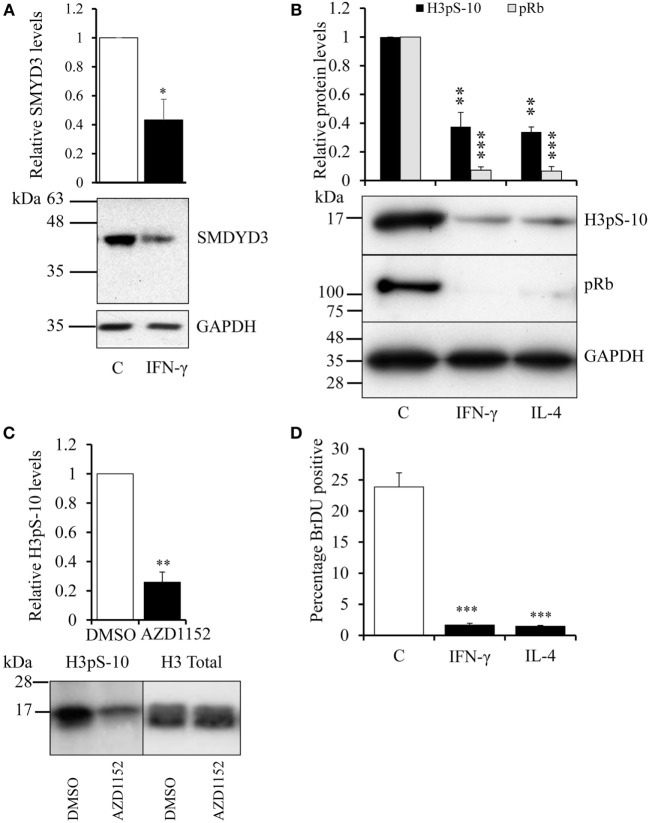
**Effect of IFN-γ, interleukin-4 (IL-4) or AZD1152 on SMYD3 protein and AURKB activity**. Four-day differentiated human blood monocyte-derived macrophages were treated with IFN-γ or IL-4 for 24 h or as indicated. Extracts were prepared for Western blotting of **(A)** SMYD3, **(B)** Histone H3-S10 phosphorylation (H3pS-10), and phosphorylated retinoblastoma protein and the densitometry results were expressed relative to untreated control. **(C)** H3pS-10 after treatment with 100 nM AZD1152, a pharmacological inhibitor of AURKB, or vehicle (DMSO) for 8 h. **(D)** 10 µM BrdU was added for a further 24 h and the percentage of nuclear BrdU positive cells was determined by immunocytochemistry and counting. Data are the mean ± SEM, *n* = 3 blood donors. *p* Values were calculated using a paired or a single value *t*-test as appropriate. *indicates *p* < 0.05, **indicates *p* < 0.01, ***indicates *p* < 0.001 compared with 24 h control.

To identify optimal time points for transcriptomic analysis, we measured changes in the mRNA expression of phenotypic markers SOCS3 (23) and CD206 (12). As expected, IFN-γ but not IL-4 induced SOCS3 mRNA expression (Figure 1A), which was significant by 6 h and remained maximal at 18 and 32 h: it then declined but was still significantly elevated above untreated controls at 48 h (Figure 1A). Also as expected, IL-4 stimulated but IFN-γ inhibited CD206 mRNA expression after 48 h (Figure 1B). Interestingly, however, IFN-γ and IL-4 both induced CD206 to a similar extent at 6 h but the effect of IL-4 increased further from 18-48 h, whereas that of IFN-γ waned such that CD206 expression had declined below control levels after 32 and 48 h (Figure 1B). We, therefore, chose the 18 h and 48 h samples from these experiments to capture the differential effects of IFN-γ and IL-4 on steady-state mRNA levels of 84 epigenetic regulators using a commercially available RT-qPCR array. The combination of IFN-γ and IL-4 was also investigated because antagonistic interactions might enhance phenotypic differences (see Introduction). The results were normalized against a panel of five housekeeping genes integral to the profiler and significant changes were identified by using the array manufacturer’s software, which yielded values of mean fold change and *p* values after false discovery rate correction (Table 2). Eight significant differences and three non-significant trends that were subsequently found significant by conventional RT-qPCR are highlighted in bold. There were more significant responses to IFN-γ (10 gene changes at 18 h, of which 7 persisted at 48 h) than IL-4 (7 gene changes, only 2 of which were significant at both 18 h and 48 h). Contrary to our expectation of antagonistic responses, IFN-γ and IL-4 appeared to produce additive effects on the expression of epigenetic regulators (Table 2). The significant changes from the RT-qPCR screen were validated and extended by a detailed time course study using the full set of samples (Figures 2A–C and 3A–H). All the significant changes observed in the array were confirmed; and also the trends toward increase of KDM6B by IL-4 and decrease of KAT2A and SETD6 by IFN-γ were shown to be significant by standard RT-qPCR normalized against total RNA, which meets current recommendations (24). Only three genes, namely CIITA, KDM6B, and NCOA1 showed increased expression (Figures 2A–C), whereas the other 8, namely AURKB, ESCO2, KAT2A, PRMT7, SETD6, SMYD3, SUV39H1, and WHSC1 showed decreased expression (Figures 3A–H). The IFN-γ-induced increases in CIITA, KDM6B, and NCOA1 showed a similar pattern to SOCS3, with significant induction by 6 h and a tendency to decline thereafter (Figures 1A and 2A–C). The IL-4-induced increase in KDM6B was also significant by 6 h of treatment but then declined, unlike CD206, which remained elevated (Figures 1B and 2B). The decreased expression of AURKB, ESCO2, KAT2A, PRMT7, SETD6, SMYD3, SUV39H1, and WHSC1 was delayed to 18 h and beyond (Figures 3A–H). In the few cases, where it was possible, we sought to confirm the mRNA data by protein or activity measurements. In the case of SMYD3, IFN-γ significantly reduced protein levels after 48 h (Figure 4A). Either IFN-γ or IL-4 inhibited AURKB activity measured by the phosphorylation of histone3 on serine-10 (H3pS-10) (Figure 4B) (25). Consistent with this, we demonstrated that AZD1152, a pharmacological inhibitor of AURKB (26) also inhibited H3pS-10 to the same extent as either IFN-γ or IL-4 (Figure 4C).

**Table 2:** **IFN-γ- or interleukin-4 (IL-4)-induced changes in expression of histone-modifying enzymes**.

		IFN-γ		IL-4		IFN-γ + IL-4	
		
		Fold change		Fold change		Fold change	
Function	Gene	18 h	48 h	18 h	48 h	18 h	48 h
Kinase	AURKB	−20.84*	−70.52*	−10.48*	−4.05*	−27.56*	−86.02*
Acetyltransferase	CIITA	11.06**		2.59*		11.32*	
	ESCO2	−56.95**	−160.89**	−15.88**	−4.59**	−62.6**	−83.09**
	KAT2A	−2.51*	**−2.3295^**			−2.3*	−3.64*
	NCOA1	2.48***	2.94**			2.58**	2.16**
Demethylase	KDM6B	2.83**	3.05 NS	**2.31^#^**		3.63**	2.9*
Methyltransferase	PRMT7	−2.23**	−2.39**			−2.26*	−3.36**
	SETD6	**−4.0798^♦^**		−2.54 NS	−2.89*	−4.94*	−5.16*
	SMYD3	−2.52*	−2.54*			−3.41*	−4.94**
	SUV39H1	−5.65**	−3.45*	−3.49**	**−**2.07 NS	−6.11**	−5.16*
	WHSC1	−2.54*	−2.46*		−2.03*	−3.7*	−2.41*

*Four-day differentiated human monocyte-derived macrophages were treated with 100 ng/mL IFN-γ or 10 ng/mL IL-4 singly or in combination for 18 h or 48 h. Extracted RNA samples were subjected to analysis by the Human Epigenetic Chromatin Modification Enzymes RT^2^ Profiler PCR Array (QIAGEN). *p* Values were calculated based on full plate normalization with twofold change as a cut off value (hence the blank values) and using a Student’s *t*-test of the replicate 2^(-Delta Ct)^ values for each gene in the control group and treatment groups*.

*indicates *p* < 0.05, **indicates *p* < 0.01, ***indicates *p* < 0.001, ^#^*p* = 0.0502, ^*p* = 0.055, **^♦^***p* = 0.054, NS indicates non-significant fold changes, *n* = 3 donors.

*AURKB, aurora kinase B; CIITA, class II major histocompatibility complex transactivator; ESCO2, Establishment of Sister Chromatid Cohesion *N*-Acetyltransferase 2; KAT2A, lysine acetyltransferase 2A, PCAF-B; NCOA1, nuclear receptor coactivator 1, Steroid Receptor Coactivator-1 (SRC-1); KDM6B, Lysine Demethylase 6B, JMJD3; PRMT7, Protein Arginine Methyltransferase 7; SETD6, SET Domain Containing 6; SMYD3, SET And MYND Domain Containing 3; SUV39H1, Suppressor Of Variegation 3-9 Homolog 1; WHSC1, Wolf-Hirschhorn Syndrome Candidate 1*.

### Decline in Histone-Modifying Enzyme Expression Was Concurrent with Decreased Proliferation

Interestingly, phosphorylation of H3pS-10 by AURKB is necessary for chromatin reorganization during mitosis (27–29), ESCO2 promotes sister chromatid cohesion (30), SUV39H1 also has a role in chromosome segregation (31, 32) and WHSC1 has been ascribed a role in DNA repair during replication (33). Hence all these enzymes have established roles in cell division. Consistent with other previous literature (34, 35), we found that IFN-γ or IL-4 inhibited phosphorylation of retinoblastoma protein (Rb, Figure 4B), confirming that they arrested cells at the G1/S checkpoint in the cell cycle (36). Treatment with either IFN-γ or IL-4 also profoundly decreased BrdU incorporation as a marker of DNA replication in our macrophages (Figure 4D). These observations led us to question whether decreased expression of histone-modifying genes by IFN-γ or IL-4 might be the incidental consequence of inhibiting proliferation.

### Growth Factor Depletion Causes a Decline in Proliferation and AURKB and ESCO2 Expression

**Figure 5:**
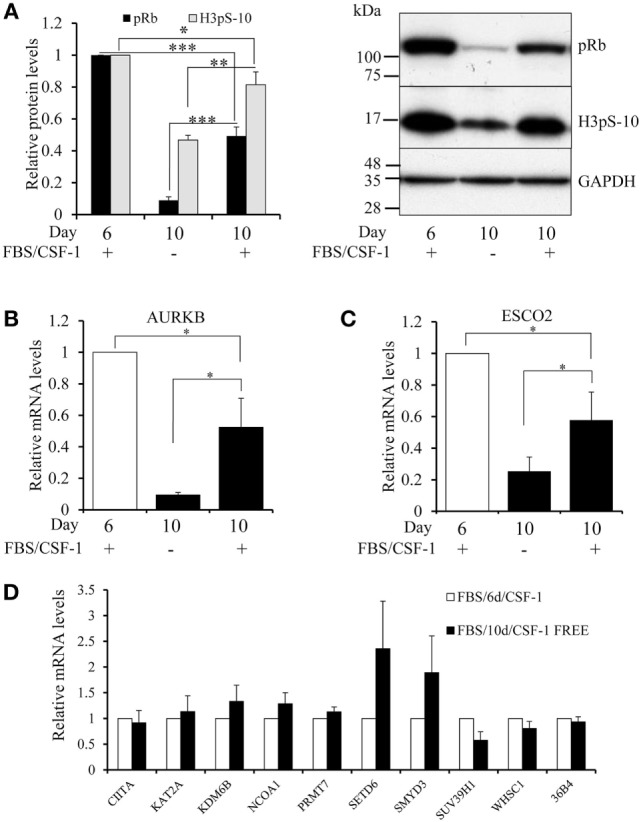
**Effect of growth factor depletion on the proliferation of macrophages, AURKB activity and mRNA levels of AURKB, ESCO2, SUV39H1, and WHSC1**. Human blood monocytes were differentiated in 10% FBS and CSF-1 for 6 days. On day 6, cells were either extracted for Western blotting and mRNA isolation or kept in culture until day 10 in either serum-free RPMI 1640 or serum and CSF-1 supplemented media before extraction. **(A)** Levels of phosphorylated retinoblastoma protein and AURKB activity (H3pS-10) were measured by Western blotting. Levels of mRNA relative to day 6 differentiated macrophages were determined by RT-qPCR for **(B)** AURKB, **(C)** ESCO2, **(D)** other genes as indicated. Data are the mean ± SEM, *n* = 3 blood donors. *p* Values were calculated using an ANOVA with Student–Newman–Keuls post-test. *indicates *p* < 0.05, **indicates *p* < 0.01, ***indicates *p* < 0.001.

To investigate the relationship between proliferation and mRNA levels of epigenetic regulators further, we cultured macrophages differentiated for 6 days for four more days either with or without growth factors, which arrests cells in the early G1 phase of the cell cycle. Consistent with this, prolonged culture decreased phosphorylated retinoblastoma protein (pRb) and H3pS-10 levels and these effects were even greater without growth factors (Figure 5A). Furthermore, BrdU incorporation after 10 days of differentiation declined by a further 76% (*n* = 4, *p* = 0.002) when growth factors were omitted. Similarly, expression of AURKB and ESCO2 mRNA each declined significantly between 6 and 10 days of culture and the decrease was greater after growth factor depletion (Figures 5B,C), suggesting that their expression was at least partly dependent on proliferation. Growth factor depletion did not affect IFN-γ or IL-4 upregulated genes, CIITA, KDM6B, or NCOA1, or the downregulated genes KAT2A, PRMT7, SETD6, or SMYD3 (Figure 5D). SUV39H1 and WHSC1 expression showed non-significant trends toward reduction (Figure 5D).

### Inhibition of Proliferation by Overexpressing p27^kip1^ Causes a Profound Decline in AURKB, ESCO2, SUV39H1, and WHSC1 Expression

**Figure 6:**
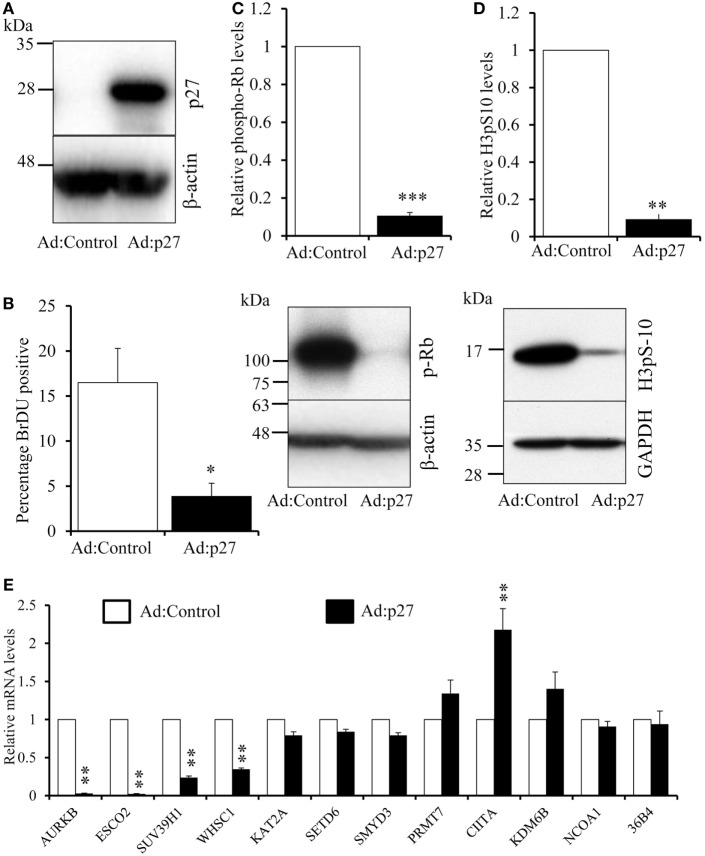
**Effect of overexpression of the cyclin-dependent kinase inhibitor p27^Kip1^**. Human blood monocytes differentiated for 4 days were infected with a recombinant adenovirus overexpressing p27^kip1^ (Ad:p27) or control, destabilized, enhanced green fluorescent protein at 1 × 10^8^ plaque forming units/ml for 24 h. **(A)** p27^kip1^ protein overexpression by Western blotting. **(B)** BrdU was added 24 h after adenovirus infection in fresh medium for further 24 h and proliferation measured as percentage BrdU positive cells using immunocytochemistry. **(C)** A representative Western blot and relative levels of phosphorylated retinoblastoma protein. **(D)** A representative Western blot and relative levels H3pS-10. *p* Values were calculated using paired or single value *t* test. *indicates *p* < 0.05, **indicates *p* < 0.01 and ***indicates *p* < 0.001 compared with Ad:control. **(E)** Effect of p27 overexpression on the mRNA levels of genes regulated by IFN-γ and interleukin-4. *p* Values calculated using an ANOVA with Dunnett post-test, **indicates *p* < 0.01 compared with Ad:control. Data are presented as the mean ± SEM, *n* = 3 blood donors.

IFN-γ or IL-4 arrest cell proliferation at the G1/S checkpoint thanks to elevation of the cyclin-dependent kinase inhibitor p21^Cip1^ (34, 35). We sought to inhibit proliferation at the G1/S checkpoint by an alternative mechanism. Given that gene silencing is inefficient in macrophages, we overexpressed the cyclin-dependent kinase inhibitor p27^kip1^ (37) from an adenovirus. Infection with the p27^kip1^ expressing virus increased p27^kip1^ protein, as expected (Figure 6A), and dramatically decreased BrdU incorporation (Figure 6B), pRb (Figure 6C) and H3pS-10 (Figure 6D). Hyperphosphorylation of Rb releases the S-phase transcription factor, E2F, which induced multiple genes that include Cyclin E and proliferating cell nuclear antigen (PCNA) (38). As expected, therefore, overexpression of p27^kip1^ profoundly decreased mRNA levels of cyclin E (84 ± 2%) and PCNA (86 ± 6%, both *n* = 3, *p* < 0.01), confirming G1/S blockade. Overexpression of p27^kip1^ decreased mRNA levels of AURKB, ESCO2, SUV39H1, and WHSC1 (Figure 6E), to a similar extent as IFN-γ or IL-4. By contrast, levels of the other genes downregulated by IFN-γ, namely KAT2A, PRMT7, SETD6, and SMYD3 were not significantly reduced by p27^kip1^ overexpression (Figure 6E) and were, therefore, clearly independent of inhibition of proliferation by either growth factor depletion or p27^kip1^ overexpression. Steady-state mRNA levels of the genes upregulated by IFN-γ or IL-4, namely CIITA, KDM6B, and NCOA1, were not decreased or even increased by overexpression of p27^kip1^ (Figure 6E).

### Overexpression of E2F1 Rescued the Downregulation of AURKB, ESCO2, SUV39H1, and WHSC1

**Figure 7:**
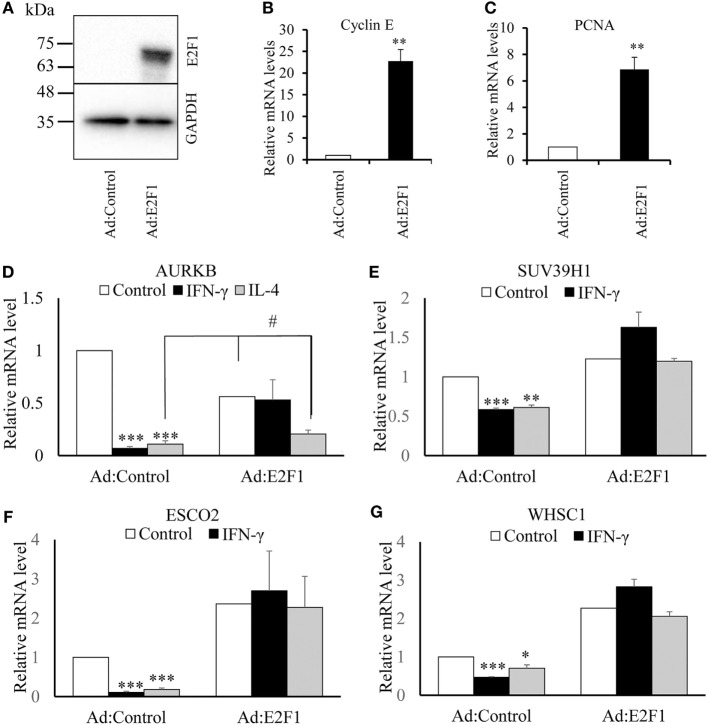
**Effect of overexpressing transcription factor E2F1**. Human blood monocytes differentiated for 4 days were infected with a recombinant adenovirus overexpressing transcription factor E2F1 or control destabilized, enhanced green fluorescent protein at 5 × 10^7^ plaque forming units/ml for 20 h. IFN-γ or interleukin-4 (IL-4) were then added for 18 h in fresh medium. **(A)** Overexpression of E2F1 protein was determined using Western blotting. **(B)** Levels of Cyclin E mRNA or **(C)** proliferating cell nuclear antigen mRNA were quantified using RT-qPCR. Concentrations of mRNA for **(D)** AURKB, **(E)** ESCO2, **(F)** SUV39H1, and **(G)** WHSC1 are expressed relative to untreated Ad:control. *p* Values were calculated using ANOVA with Student–Newman–Keuls post-test. *indicates *p* < 0.05, **indicates *p* < 0.01, ***indicates *p* < 0.001, compared with Ad:control ^#^indicates *p* < 0.05 compared with Ad:control + IL-4 and AdE2F1 alone. Data are presented as the mean ± SEM, *n* = 4 blood donors.

It has been suggested that AURKB is a direct target of transcription factor E2F (29). Furthermore, when we interrogated the ENCODE database, we found chromatin immunoprecipitation evidence for binding of E2F transcription factors to the proximal promoters of the AURKB, ESCO2, SUV39H1, and WHSC1 genes, whereas CIITA, KDM6B, and NCOA1 had no such sites. We, therefore, investigated whether the effects of IFN-γ or IL-4 on AURKB, ESCO2, SUV39H1, and WHSC1 could be reversed by adenovirus-mediated overexpression of E2F1. Infection with the E2F1 expressing virus increased E2F1 protein (Figure 7A) and, as expected, the mRNA levels of the known E2F responsive genes, Cyclin E and PCNA (38) (Figures 7B,C). E2F1 gene transfer completely reversed the inhibitory effect of IFN-γ and IL-4 on AURKB, ESCO2, SUV39H1, and WHSC1 mRNA levels (Figures 7D–G), except in the case of AURKB for which the effect of IL-4 was only partly reversed (Figure 7D). These data provided further support for the conclusion that the effects of IFN-γ or IL-4 on these genes were, at least partly, mediated indirectly through inhibition of proliferation.

### Impact of Pharmacological Inhibition and or shRNA Silencing of KDM6B on Macrophage Polarization

**Figure 8:**
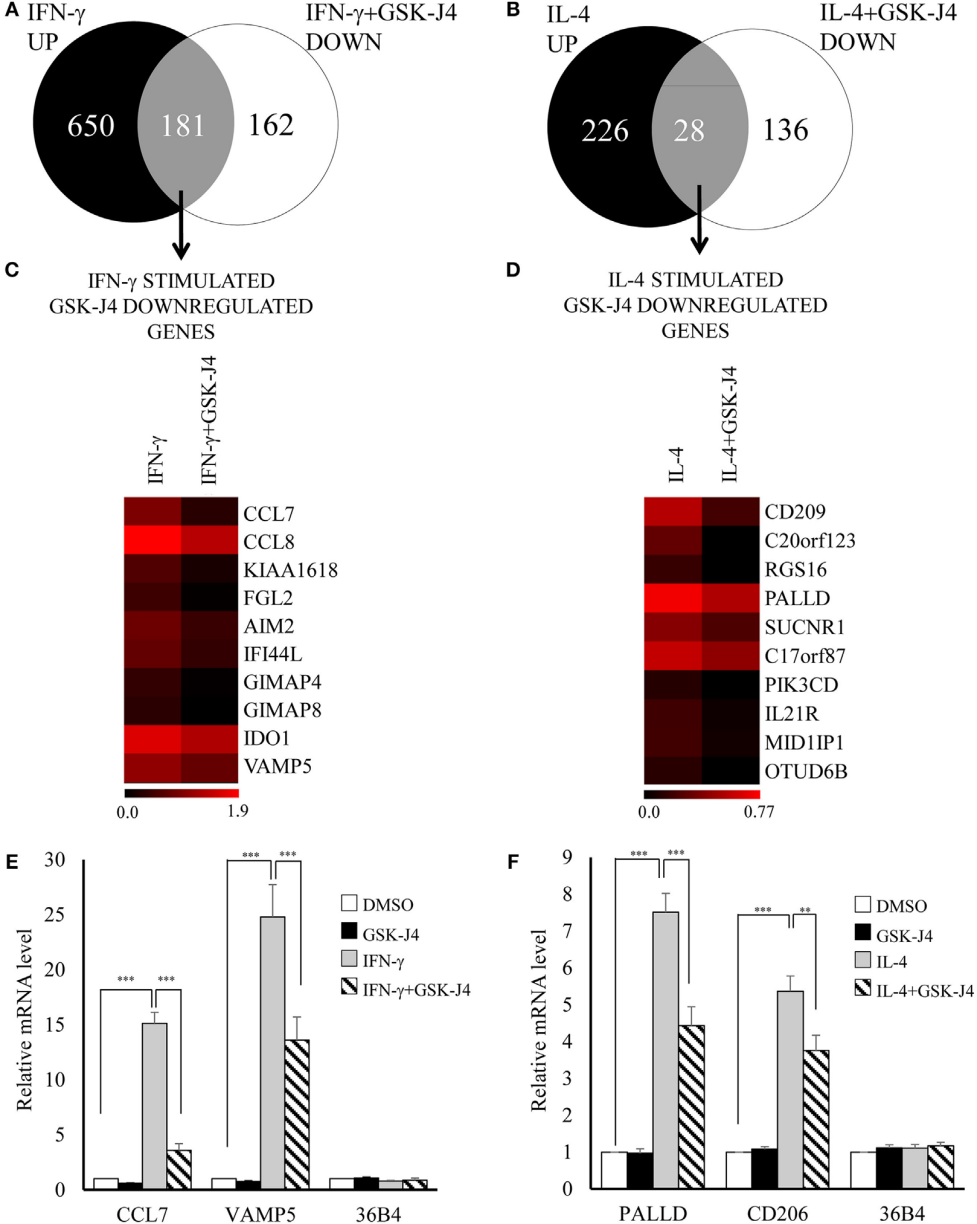
**Effect of KDM6 inhibitor, GSK-J4, on the transcriptome of IFN-γ or interleukin-4 (IL-4) stimulated macrophages**. Human blood monocytes differentiated for 4 days were pre-treated with 60 µM GSK-J4 or vehicle (DMSO) for 0.5 h and were then treated with either 100 ng/mL of IFN-γ or 10 ng/mL of IL-4 for further 6 h. Purified RNA samples (*n* = 4 donors) were analyzed on the Illumina Human HT-12v4 microarray or by standard RT-qPCR. Venn diagrams summarizing changes in response to **(A)** GSK-J4 ± IFN-γ or **(B)** GSK-J4 ± IL-4. Heat maps of 10 genes inhibited by GSK-J4 more than twofold that were upregulated by **(C)** IFN-γ or **(D)** IL-4 [the scale is log(fold change)]. Validation of selected changes in response to **(E)** GSK-J4 ± IFN-γ or **(F)** GSK-J4 ± IL-4 using RT-qPCR. Data are presented as the mean ± SEM for *n* = 4 blood donors. **indicates *p* < 0.01, ***indicates *p* < 0.001 vs IFN-γ or IL-4 alone.

**Figure 9:**
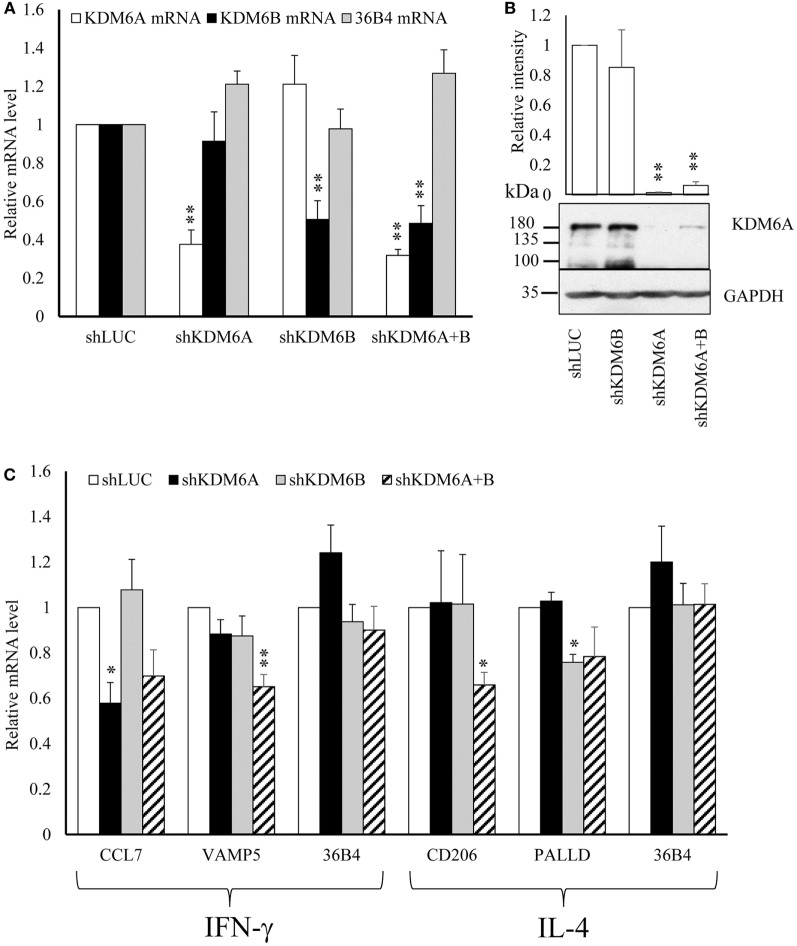
**Effect of shRNA silencing of KDM6A and KDM6B**. Human blood monocytes differentiated for 4 days were infected with 2 × 10^8^ pfu/ml of each individual shRNA adenovirus for 72 h and were then treated with no addition, 100 ng/mL of IFN-γ, or 10 ng/mL of interleukin-4 (IL-4) for further 6 h. **(A)** The levels of mRNAs for KDM6A, KDM6B, or housekeeping gene, 36B4, were measured in cells infected with adenovirus expressing shKDM6A or shKDM6B were normalized against those with shLuciferase (shLUC) as control. **(B)** Protein of levels KDM6A and housekeeping gene GAPDH under the same conditions. **(C)** The effects of shKDM6A, shKDM6B, individually, or together on mRNA levels of genes upregulated by IFN-γ or IL-4. Data are presented as the mean ± SEM relative to shLUC for *n* = 6 blood donors. *indicates *p* < 0.05, **indicates *p* < 0.01 vs shLUC.

From the above results we concluded that increased expression of CIITA, KDM6B, and NCOA1 and decreased expression of KAT2A, PRMT7, SETD6, and SMYD3 was independent of any effects on proliferation. To begin to understand the impact of these changes on macrophage phenotype, we chose to further investigate KDM6B, which was the only transcriptional enhancer that was increased at the mRNA level by both IFN-γ and IL-4. Given that the action of KDM6B is demethylation of lysine 27 on histone3, which is associated with increased gene transcription (13), this upregulation might be expected to promote transcriptional responses to both cytokines. Conversely, inhibition and silencing of KDM6B might decrease gene expression associated with IFN-γ and IL-4. To narrow down the search for those genes regulated by KDM6B, either on its own or in combination with KDM6A, we first performed a microarray study of transcripts upregulated by IFN-γ or IL-4 in the presence and absence of the combined KDM6A and B inhibitor GSK-J4 (39). From preliminary time course and dose-response studies (results not shown), 6 h exposure was sufficient and 60 µM GSK-J4 was chosen because it significantly suppressed TNFα induction by lipopolysaccharide (LPS) by approximately 70% [confirming previous results (39)]. CD206 induction by IL-4 was also inhibited, albeit by only 30% (both *p* < 0.05), whereas a housekeeping gene, 36B4, was not affected. From these pre-validated samples, the transcriptomic analysis showed that IFN-γ significantly upregulated 906 and IL-4 upregulated 271 transcripts after 6 h (a complete gene list is deposited under GSE83957). Only 62 of these (6%) were upregulated by both IFN-γ and IL-4, which confirms the differential phenotypes stimulated by these two cytokines, as demonstrated more extensively previously (1). This data also emphasizes how unusual KDM6B is in being upregulated by both IFN-γ and IL-4. Of the 831 IFN-γ upregulated transcripts recognized by the genevenn program used to generate Venn diagrams, 181 (22%) were significantly decreased by the additional presence of GSK-J4 (Figure 8A). Using GOE and KEGG pathway analysis, these genes were associated with several aspects of immune cell function and transcriptional activation (Tables 3 and 4). Of the 254 IL-4 upregulated transcripts only 28 (11%) were significantly reduced by the additional presence of GSK-J4 (Figure 8B). There were insufficient genes in this cluster to perform GOE or KEGG pathway analysis. Only two transcripts (C17orf87 and LOC650919), neither associated with a known function, were common to both lists. The genes most inhibited by GSK-J4 in the presence of either IFN-γ or IL-4 are illustrated by heat maps in Figures 8C,D, respectively, with the details of these and further genes listed in Table 5. Heading the list of IFN-γ upregulated, GSK-J4 downregulated genes were CCL7 and CCL8, which are known genes associated with activation by IFN-γ (40). Heading the list of IL-4 upregulated, GSK-J4 downregulated gene was CD209, which is an established IL-4 responsive gene (41). A selection of the more abundant transcripts that were inhibited at least twofold by GSK-J4, were chosen for further analysis. Because there were so few abundant IL-4 stimulated, GSK-J4 inhibited transcripts in the array, we also included CD206, which was used for initial sample validation but just failed to reach significance in the array experiment (i.e., a false negative). First, upregulation by IFN-γ or IL-4 and its reversal by GSK-J4 was confirmed by RT-qPCR (Figures 8E,F, respectively). GSK-J4 is non-selective for KDM6A and KDM6B (39). Hence, to distinguish the roles of KDM6A and KDM6B, the effects of silencing one, the other or both was investigated by using shRNA. Given that silencing is difficult in primary macrophages, we used adenovirus-mediated delivery of shRNAs selective for KDM6A or KDM6B and compared these to delivery of a control adenovirus that expressed shRNA against firefly luciferase (shLUC). The housekeeping gene, 36B4, was also studied as a further control. Based on mRNA levels, silencing of KDM6A and B was highly selective, albeit incomplete at the maximum tolerable adenovirus dose (Figure 9A). Specificity and efficacy was confirmed at the protein level for KDM6A (Figure 9B) but no suitable antibody is available for KDM6B. The effects of KDM6A and B silencing were measured in the presence of IFN-γ or IL-4. From these results, the induction of CCL7 by IFN-γ depended selectively on KDM6A, whereas that of VAMP5 required KDM6A and B redundantly (Figure 9C). The induction of CD206 by IL-4 also required KDM6A and B redundantly, whereas that of PALLD depended selectively on KDM6B (Figure 9C). We concluded that a subgroup of transcripts upregulated by IFN-γ or IL-4 depended on KDM6B, either on its own or redundantly with KDM6A.

**Table 3:** **The most significantly enriched Gene Ontology Enrichment (GOE) terms of IFN-γ-stimulated GSK-J4-inhibited genes**.

GOE	Term description	Genes in term	% of genes in term	*p*-Value	Fold enrichment
GO:0006955	Immune response	32	18.9	4.90E−13	4.71
GO:0009615	Response to virus	15	8.87	2.60E−12	13.99
GO:0006952	Defense response	25	14.79	5.20E−09	4.13
GO:0002697	Regulation of immune effector process	11	6.50	5.00E−08	11.07
GO:0002821	Positive regulation of adaptive immune response	7	4.14	4.60E−07	23.73
GO:0002684	Positive regulation of immune system process	14	8.28	5.60E−07	5.98
GO:0048584	Positive regulation of response to stimulus	13	7.69	3.30E−06	5.60
GO:0050865	Regulation of cell activation	11	6.50	8.10E−06	6.39
GO:0001817	Regulation of cytokine production	11	6.50	1.10E−05	6.18
GO:0002694	Regulation of leukocyte activation	10	5.91	3.50E−05	6.12

*GOE analysis was performed by using the DAVID public database (https://david.ncifcrf.gov/)*.

**Table 4:** **The most significantly enriched KEGG pathways of IFN-γ-stimulated GSK-J4 inhibited genes**.

KEGG	KEGG pathway	Genes in pathway	% of genes in pathway	*p*-Value	Fold enrichment
hsa04062	Chemokine signaling pathway	8	4.73	0.00	3.29
hsa04672	Intestinal immune network for IgA production	4	2.36	0.02	6.28
hsa04630	Jak-STAT signaling pathway	6	3.55	0.04	2.98

*KEGG pathway analysis was performed by using the DAVID public database (https://david.ncifcrf.gov/)*.

**Table 5:** **Genes significantly upregulated by IFN-γ or interleukin-4 (IL-4) and most downregulated by GSKJ-4**.

Gene	IFN-γ + DMSO vs DMSO	IFN-γ + GSK-J4 vs IFN-γ + DMSO	Gene	IL-4 + DMSO vs DMSO	IL-4 + GSK-J4 vs IL-4 + DMSO
CCL7	8.66	−4.33	CD209	3.53	−2.2
CCL8	82.8	−3.6	C20orf123	1.99	−2.07
KIAA1618	4.1	−2.71	RGS16	1.46	−1.81
FGL2	2.82	−2.59	PALLD	5.44	−1.59
AIM2	6.4	−2.37	SUCNR1	2.55	−1.49
IFI44L	5.53	−2.28	C17orf87	3.91	−1.45
GIMAP4	2.42	−2.24	PIK3CD	1.28	−1.41
GIMAP8	2.13	−2.24	IL21R	1.55	−1.4
IDO1	43.61	−2.19	MID1IP1	1.59	−1.38
VAMP5	12.01	−2.09	OTUD6B	1.32	−1.37
MX2	2.92	−2.08	DAAM1	1.64	−1.36
ST3GAL5	1.36	−2.05	PLEKHF2	1.3	−1.32
CBX6	1.86	−2	HOPX	1.72	−1.3
KCNJ2	1.75	−1.934	METTL7A	1.26	−1.28
GIMAP7	2.6	−1.933	PIK3R6	1.42	−1.28
FST	2.34	−1.92	LOC729222	1.37	−1.27
ANKRD22	7.6	−1.9	PLA2G4A	1.23	−1.26
TNFSF10	7.45	−1.89	PPFIBP1	1.25	−1.24
PPP2RB	2.42	−1.87	CISH	2.66	−1.22
IFI44L	3.64	−1.78	C14orf149	1.22	−1.21
GIMAP6	1.8	−1.77	IL1RAP	1.33	−1.19
ISG15	2.7	−1.71	LOC100129269	1.54	−1.19
GBP1	17.95	−1.7	EFNA1	1.75	−1.19
P2RY14	3.47	−1.69	PHOSPHO1	1.16	−1.16
F3	3.06	−1.65	TMEM39B	1.16	−1.15
ENPP2	2.32	−1.65	PBX2	1.28	−1.14
GIMAP5	2.2	−1.64	EXOSC4	1.14	−1.14
STAMBPL1	4.13	−1.6376	LOC650919	1.17	−1.13
APOBEC3G	2.19	−1.6374			
STAT1	5.19	−1.63			
CD97	1.79	−1.62			
LOC400759	7.91	−1.61			
ARID5B	2.5	−1.61			
C17orf87	2.06	−1.59			
GBP2	6.61	−1.58			
UGDH	3.03	−1.57			
LOC728855	3	−1.56			
ASAP2	1.31	−1.554			
TMEM194A	2.62	−1.551			

*Four-day human monocyte-derived macrophages were treated with IFN-γ or IL-4 in the presence of DMSO vehicle or 60 µM GSK-J4 with DMSO alone as control. Fold changes in mRNA expression from the Illumina HumanHT-12 v4 microarray were calculated using GeneSpring. *p* Values calculated using the Benjamini–Hochberg false discovery rate for multiple testing correction were in all cases <0.05 (*n* = 4 separate donors)*.

## Discussion

### Main Findings

Using a focused array, we demonstrated that IFN-γ or IL-4 modulate the mRNA expression of at least seven epigenetic regulators in human blood monocyte-derived macrophages, independently of any effects on cell proliferation. CIITA, KDM6B, and NCOA1 showed increased mRNA expression within 6 h of stimulation, whereas KAT2A, PRMT7, SETD6, and SMYD3 showed decreased expression that required at least 18 h of treatment. The effects were confirmed at the level of protein for SMYD3. Based on similar effects of growth factor depletion or overexpression of p27^kip1^, we concluded that IFN-γ or IL-4 decreased expression of AURKB, ESCO2, SUV39H1 and WHSC1 mRNA and AUKB activity, at least partly, as a consequence of cell cycle arrest at the G1/S checkpoint. Furthermore, this decreased expression could be reversed by overexpression of E2F1, which is known from published chromatin immunoprecipitation (ENCODE) studies to bind directly to the relevant promoter regions. These data expand the list of epigenetic regulators the expression of which is regulated by IFN-γ and IL-4. As a first step to establishing whether these changes impact on macrophage phenotype, we demonstrated by pharmacological inhibition and shRNA silencing that KDM6B participates in a subset of the divergent gene expression changes in response to IFN-γ and IL-4.

### Role of Diverse Epigenetic Regulators

In general, several families of enzymes that can alter the phosphorylation, acetylation, and methylation status of specific histone residues play a major role in epigenetic regulation (42–44). Histone H3S-10 phosphorylation catalyzed by AURKB has been implicated in chromatin condensation during mitosis (45, 46). Moreover, the finding that AURKB is an E2F target (29) led to the conclusion that it is a useful marker of cell proliferation, similar to PCNA. Our data showing that AURKB is downregulated by IFN-γ and IL-4-induced cell cycle arrest and is restored by the E2F1 extend these conclusions to primary macrophages. Previous work showing that the AURKB inhibitor, AZD1152, abrogates growth of human acute myeloid leukemia cells (47) and that growth arrest of mouse Raw264.7 macrophages by *H. Pylori* is associated with downregulation of AURKB (48) are also consistent with our conclusions.

Histone acetyl transferases (HATs) promote opening of the chromatin and enhance transcription, whereas histone de-acetylases (HDACs) have the opposite effect (49–51). The ability of CIITA to recruit HATs, including KAT2A (also known as pCAF-B) and NCOA1 (also known as SRC-1), to the promoter of the major histocompatibility complex-II (MHC-II) gene, has been extensively studied in macrophages (52, 53). Given the previous literature, CIITA upregulation by IFN-γ could be seen as a positive control for our array study. However, we also found CIITA to be upregulated to a lesser extent by IL-4, and both effects were independent of cell proliferation. Upregulation of NCOA1 selectively by IFN-γ most likely enhances the effects CIITA (52, 53) but downregulation of KAT2A seems paradoxical. However, this might also enhance the action of IFN-γ by decreasing acetylation and potentiating the functions of IRFs (54). The HAT, ESCO2, was also downregulated by IFN-γ or IL-4, although from our data this appeared to be mainly the consequence of the inhibition of cell proliferation. As its full name “Establishment of Sister Chromatid Cohesion *N*-Acetyltransferase” implies, ESCO2 has a known role in mitosis (30). It functions as part of the cohesion complex and its mutation leads to the cohesinopathy, Roberts syndrome (55). However, cohesin (and perhaps therefore ESCO2) has also been ascribed a wider role in gene transcription (56–58) and ESCO2 participates in Notch signaling (59), observations that might have implications for proliferating macrophages, although this remains to be investigated. Overall, our results imply that treatment with IFN-γ has the ability to both increase and decrease activity of specific HATs, thereby increasing or decreasing expression of different genes. In future experiments, beyond the present scope, it will be interesting to investigate the effects of manipulating levels of the HATs we have identified as up- or downregulated on both positive and negative transcriptional responses to IFN-γ. Recent data from the group of de Winther and colleagues showed, for example, that IFN-γ treatment specifically altered the acetylation status of the promoters of two downregulated genes, *Il1b* and *Il6*, in mouse macrophages (60), although the role of specific HATs and HDACs in these changes was not defined. We did not detect effects of IFN-γ or IL-4 on mRNA expression of any of the HDACs-1 to -11 that were included in our RT-qPCR screen. However, other mechanisms including changes in recruitment and activation of HATs and HDACs at specific promoters also contributes to acetylation status (61).

Histone methyl transferases (HMTs) and lysine de-methylases (KDMs) can be stimulatory or inhibitory to transcription depending on the site and degree of methylation that is optimal (62). For example, H3K4 methylation has important consequences for both enhancer and promoter activity of macrophage specific genes (63). Mono and di-methylation appear permissive for enhancers but tri-methylation for promoters of LPS-sensitive genes (61). A previous study on human macrophages stimulated with LPS and IFN-γ (64) showed increased H3K4 methylation associated with increased expression of the HMT, myeloid lymphoid leukemia (MLL). Given that we did not see any change in MLL expression with IFN-γ alone (absent from Table 2), it is possible that this is an effect of LPS, although this requires verification. In our study, the HMTs, PRMT7, SETD6, and SMYD3, were downregulated after priming by IFN-γ alone, independently of inhibition of proliferation. PRMT7 is a member of the PRMT histone arginine methylases, whereas SETD6 and SMYD3 are lysine methyl transferases. PRMT7 upregulates expression of metalloproteinase-9 (MMP-9) (65) in breast carcinoma cells but its role in macrophages is unknown. The related PRMT4 promotes major histocompatibility II (MHCII) gene expression (52). SETD6 activity has been linked to repression of the nuclear factor κB (NF-κB) system (66, 67) and upregulation of estrogen-responsive genes (68) in other cell types but there appears to have been very little previous work in mouse or human macrophages beyond demonstrating its presence preferentially in alternatively activated human macrophages, consistent with our results (64). SMYD3 di- and tri-methylates H3K4 residues (62). Although not an S-phase gene, SMYD3 has been identified as essential for cancer cell proliferation (69). It also plays a role in rescue from senescence (70), estrogen response (71), and MMP-9 induction (72) in various cancer cell lines. SMYD3 is also little studied in macrophages, although a previous study demonstrated its downregulation by a combination of LPS and IFN-γ, which is consistent with our findings (64).

In addition, the HMTs, SUV39H1, and WHSC1, were downregulated by IFN-γ or IL-4, at least in part, as a consequence of cell cycle arrest. Since SUV39H1 methylates H3K9 and places a repressive mark (62), it is predicted to reduce transcription of susceptible genes. Interestingly, one of the genes decreased by SUV39H1 in macrophages is p21^waf1^ (73), the cyclin-dependent kinase inhibitor that is responsible for inhibition of proliferation by IFN-γ or IL-4 (34, 35). Conversely, expression of p21^waf1^ indirectly downregulates SUV39H1, which implies a mutual feedback mechanism that presumably fine tunes the rate of proliferation. WHSC1 is a candidate gene implicated in Wolf-Hirschhorn syndrome, which is caused by deletions within the chromosome 4p16.3 region (74, 75). It has the ability to methylate several lysine residues in H3 and H4 (76) and could, therefore, act as a transcriptional activator or repressor. It has been ascribed a variety of functions, including in replicative DNA repair (33), which implies a role in S phase, but also in sustaining NF-κB pathway activity in tumors (77), which suggests activity may be present in other phases of the cell cycle. So far, there appears to be no knowledge regarding its role in macrophages.

We chose to prioritize the lysine demethylase, KDM6B, for study in greater detail because it removes repressive H3K27Me3 marks and is, therefore, a putative transcriptional activator. It is also amenable to selective pharmacological inhibition, which would lead to downregulation of target gene expression. Furthermore, KDM6B has been previously implicated in macrophage polarization by either bacterial LPS or IL-4, depending on the source of macrophages investigated. For example, KDM6B is upregulated in response to bacterial LPS in both mouse (78) and human (39) macrophages; and as many as 70% of LPS responsive genes in mouse macrophages recruit KDM6B to their promoters (15). This does not always lead to H3K27 demethylation (15) but KDM6A and B nevertheless act redundantly to potentiate responses to LPS in human macrophages (39). Other work in mouse macrophages showed that KDM6B can be upregulated by IL-4 in a STAT-6-dependent manner and that it is essential for IL-4 induced polarization *in vitro* and in response to certain kinds of parasitic infection *in vivo* (14, 17). However, no previous study has investigated the role of KDM6B on both pro-inflammatory and anti-inflammatory polarization in the same preparation of macrophages. Our transcriptomic study demonstrated for the first time that KDM6A and B play a part in polarization by IFN-γ, although a smaller proportion of genes (approximately 20%) appear to be affected than for responses to LPS (39). Likewise, KDM6B modulates some IL-4 polarization genes alone or redundantly with KDM6A in human macrophages but this seems to be a much small proportion (11%) than in mouse macrophages (14, 17). The finding that KDM6A and B acted redundantly for some processes is consistent with the previous study on LPS (39).

### Implications

Our studies significantly expand knowledge of the expression changes in epigenetic regulators during polarization of human macrophages. Upregulation or downregulation of genes does not necessarily imply that these will be the only enzymes that play a major role in responses to IFN-γ or IL-4. However, the previous literature on CIITA and NCOA1 together with our new results with KDM6B illustrates the importance of upregulated genes. Importantly, we identify several changes that are independent of inhibition of proliferation, a complicating factor that appears to have been overlooked in previous studies. However, those changes partly dependent of proliferation may also be of significance. Indeed, recent work has highlighted the importance of proliferation in replenishing populations of resident macrophages (79). There have also been interesting findings in models of inflammation, especially atherosclerotic plaque formation (80), suggesting that proliferation rather than recruitment may play the major role in sustaining macrophage numbers. From our data, proliferation has a major impact on epigenetic programing, and this undoubtedly influences macrophage behavior. Future work, beyond the present scope, should probe into the downregulated genes we have identified, many of which are virtually unstudied in macrophages. However, we recognize that mechanisms other than changes in expression level regulate the function of epigenetic writers, readers and erasers at the promoters and enhancers of pro- and anti-inflammatory genes (61, 81). Except in the case of KDM6B, the contribution that the expression changes we observed make to epigenetic regulation in macrophages remains to be established.

## Author Contributions

GY-B performed and interpreted part of the experimental work and helped prepare the manuscript. MB contributed to the experimental design, interpretation of results, and writing of the manuscript. GS-N planned, performed, and interpreted the molecular biology component of the work. CH interpreted the microarray data. AN planned the study and led the interpretation of the results and writing of the manuscript.

## Conflict of Interest Statement

The authors declare that the research was conducted in the absence of any commercial or financial relationships that could be construed as a potential conflict of interest.

## Footnotes

^1^http://pcrdataanalysis.sabiosciences.com/pcr/arrayanalysis.php.

^2^http://www.embl-ebi.ac.uk/arrayexpress/experiments/E-GEOD-48977/protocols/.

^3^http://genevenn.sourceforge.net/.

^4^https://david.ncifcrf.gov/.
